# Using Ecological Niche Modeling to Predict the Spatial Distribution of *Anopheles maculipennis* s.l. and *Culex theileri* (Diptera: Culicidae) in Central Iran

**Published:** 2019-06-24

**Authors:** Najmeh Hesami, Mohammad Reza Abai, Hassan Vatandoost, Mostafa Alizadeh, Mahboubeh Fatemi, Javad Ramazanpour, Ahmad Ali Hanafi-Bojd

**Affiliations:** 1 Department of Medical Entomology and Vector Control, School of Public Health, Tehran University of Medical Sciences, Tehran, Iran; 2 Department of Environmental Chemical Pollutants and Pesticides, Institute for Environmental Research, Tehran University of Medical Sciences, Tehran, Iran; 3 Department of Communicable Diseases Control, Deputy for Health, Isfahan University of Medical Sciences, Isfahan, Iran

**Keywords:** Culicidae, Spatial distribution, *Culex theileri*, *Anopheles maculipennis* s.l, Ecological niche modeling

## Abstract

**Background:**

Mosquitoes are very important vectors of diseases to human. We aimed to establish the first spatial database on the mosquitoes of Isfahan Province, central Iran, and to predict the geographical distribution of species with medical importance.

**Methods:**

Mosquito larvae were collected from eight counties of Isfahan Province during 2014. Collected data were transferred to a database in ArcGIS and the distribution maps were created. MaxEnt model and jackknife analysis were used to predict the geographical distribution of two medical important species, and to find the effective variables for each species.

**Results:**

Totally, 1143 larvae were collected including 6 species, *Anopheles maculipennis* s.l., *An. superpictus* s.l., *An. marteri*, *Culex hortensis*, *Cx. theileri* and *Culiseta longiareolata*. The area under curve in MaxEnt model was 0.951 and 0.873 rather 1 for *An. maculipennis* s.l. and *Cx. theileri*, respectively. *Culex theileri* had wider and more appropriate niches across the province, except for the eastern area. The environmental variable with highest gain was mean temperature of the wettest quarter for *Cx. theileri* and temperature seasonality for *An. maculipennis*. *Culex theileri*, *An. maculipennis* s.l. and *An. superpictus*, three important vectors of parasitic agents to humans, were collected in this study.

**Conclusion:**

The mosquito collected and mapped can be considered for transmission of malaria and filariasis in the region. Bearing in mind the results of niche modeling for vector species, more studies on vectorial capacity and resistance status to different insecticides of these species are recommended.

## Introduction

Mosquitoes are one of the most important groups of medical arthropods and transmit malaria, filariasis, different arboviruses and encephalitis as well as annoyance due to their bites (1). A serological study in 1970s showed West Nile infection was relatively common in Iran with a prevalence of 30%, while infection with Sindbis virus was very rare (2).

*Culex theileri* is the principal epidemic vector of Rift Valley fever virus (Bunyaviridae: Phlebovirus) on the inland plateau of southern Africa (3). Dirofilariasis due to *Dirofilaria immitis* and *D. repens* and setariasis have been reported in current studies from some parts of Iran, while West Nile Virus is also detected in current studies from the birds, horse, human and mosquitoes (4–14). This is in accordance with the global trends of these diseases, although both animal and human cases of this disease are under estimated (15, 16).

Study on the mosquitoes of Iran has a long history and is conducted on different aspects such as fauna, distribution, parasitic infection, resistance to insecticides and modeling distribution (5, 6, 17–20). *Culex theileri* is reported from 27 out of 31 provinces of Iran including Ardabil, West Azarbaijan, East Azarbaijan, Bushehr, Charmahal and Bakhtiari, Fars, Guilan, Hamadan, Hormozgan, Ilam, Isfahan, Kerman, Kermanshah, South Khorassan, North Khorassan, Khorassan-e Razavi, Khuzestan, Kohgiluyeh and Boyerahmad, Kordestan, Lorestan, Markazi, Mazandaran, Qom, Sistan and Baluchestan, Tehran, Yazd, and Zanjan (21).

*Anopheles maculipennis* s.l. is one of the main malaria vectors in its distribution areas including Iran (22). It is reported from 20 provinces of Iran including Ardabil, West Azarbaijan, East Azarbaijan, Charmahal and Bakhtiari, Guilan, Golestan, Hamadan, Isfahan, Kermanshah, Khorassan-e Razavi, North Khorassan, Kohgiluyeh and Boyerahmad, Kordestan, Qazvin, Lorestan, Markazi, Mazandaran, Semnan, Tehran, and Zanjan (22).

Despite activities regarding mosquito control in Iran, there is still the problem of painful bites of these insects, especially in central areas like Isfahan, Arak, Semnan, and Tehran provinces. Due to the vital role of health in development programs, and because the study on ecology and bionomics of mosquitoes is one of the important indices that have a fundamental role in development of ecotourism industry, proper knowledge from behavioral characteristics and bionomics of mosquitoes in different ecological conditions is one of the main factors in planning the strategy to combat the mosquitoes. Isfahan Province is one of the main poles for industry and tourism in Iran. Although rare studies have been done in past (23, 24), but still there is no comprehensive survey on vector(s) bioecology in different climates, seasons, temperatures, and so on. With due attention to the extensive climate change in the world and Iran as well, accurate study on the ecology and bionomics of mosquitoes are necessary due to their important role in disease transmission, especially arboviruses. Data collection on their biodiversity, distribution and ecology will provide guideline for appropriate vector control.

Geographic Information Systems (GIS) is a rapidly growing technology that combines graphics features with the environmental data obtained from the vectors of disease. This ability helps us to assess the distribution and bioecology of vector species. In last two decades using GIS in vector-borne diseases has increased and a new field of investigation has been opened. Creating databases, mapping, spatial and statistical analysis of vector-borne diseases are results of this new branch of science (25).

This study aimed to establish the first spatial database on the mosquitoes of Isfahan Province and to predict the geographical distribution of medically important species.

## Materials and Methods

### Study Area

**Fig. 1.**
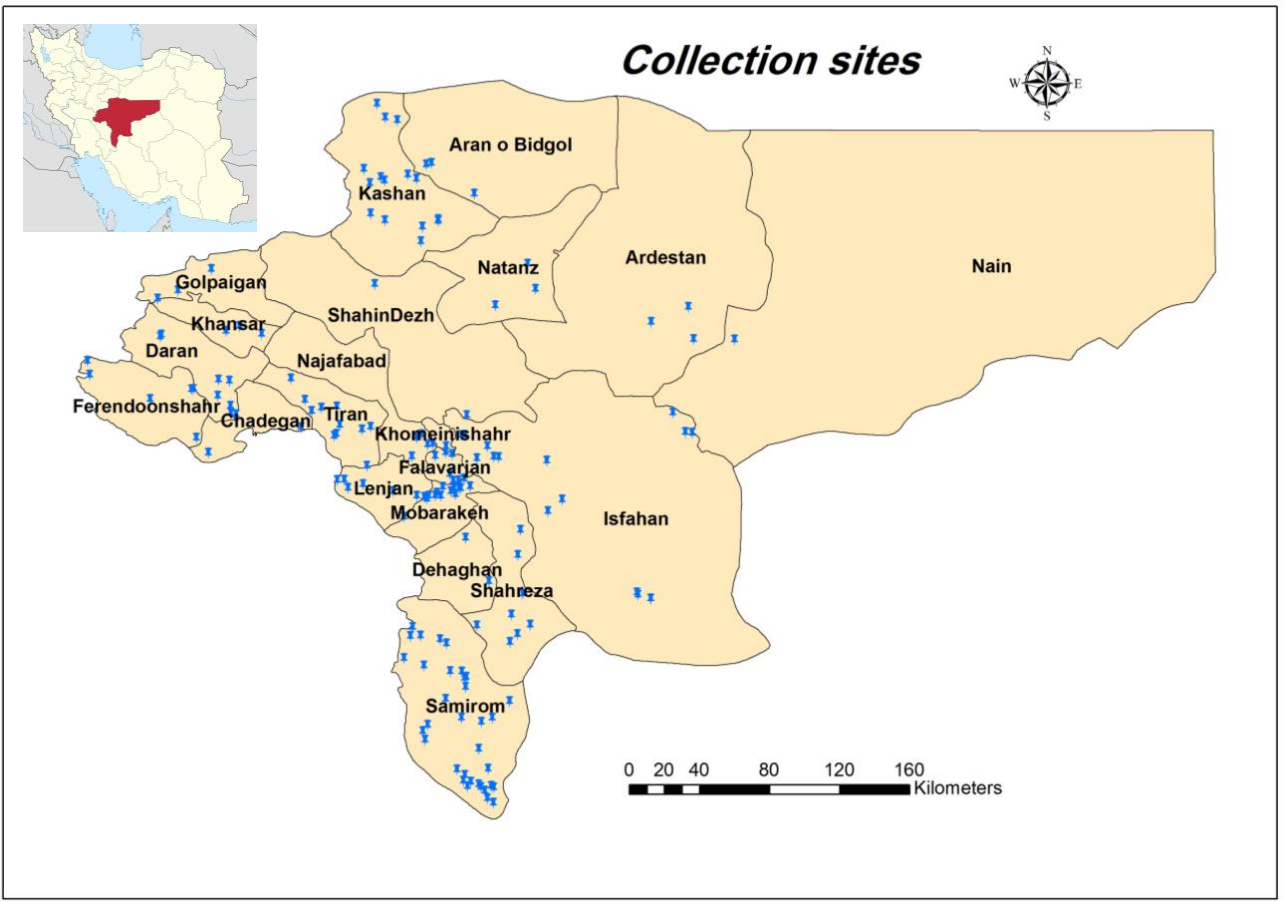
Study sites in Isfahan Province, Iran

The Isfahan Province covers an area of approximately 107,027km^2^ and is situated in the center of Iran (Fig. 1). The province experiences a moderate and dry climate, on the whole, ranging between 40.6 °C and 10.6 °C on a cold day in the winter season. The average annual temperature has been recorded as 16.7 °C and the annual rainfall on an average has been reported as 116.9mm. More than 5 million peoples are living in 24 counties of this province. Isfahan is destination of millions of tourists from different parts of Iran and other countries.

### Entomological Survey

Sampling was conducted two times during summer 2015 from 8 counties (Khomeinishahr, Golpayegan, Faridan, Khansar, Mobarakeh, Fereidoonshahr, Najafabad, Samirom) in three main topographic areas of Isfahan Province. Mosquito larvae were collected by the standard dipping method. Coordinates of the collections sites were recorded using a GPS device. Species were transferred to the laboratory, mounted and identified morphologically (26).

All the mounted slides were deposited in the Medical Entomology Museum, School of Public Health, Tehran University of Medical Sciences under code of GC22ST11-94.

### Creating Database and Mapping

All collected data obtained from this entomological survey were transferred to a relevant database in ArcGIS and then the distribution maps were created.

### Modeling

MaxEnt model and bioclimatic variables were used to predict and to map the geographical distribution of medically important species which had enough occurrence data points (27). Jackknife test in MaxEnt model was used to find the effective variables for each species. The bioclimatic data were downloaded from the worldclim database in 1km^2^ spatial resolution (version1.4, http://www.worldclim.org/past). They were included Bio1 (Annual mean temperature (^o^C), Bio 2 (Mean diurnal range: mean of monthly (max temp–min temp) (°C)), Bio3 (Isothermality: (Bio2/Bio7)× 100), Bio4 (Temperature seasonality (SD× 100)), Bio5 (Maximum temperature of warmest month (°C)), Bio6 (Minimum temperature of coldest month (°C)), Bio7 (Temperature annual range (Bio5−Bio6) (°C)), Bio8 (Mean temperature of wettest quarter (°C)), Bio9 (Mean temperature of driest quarter (°C)), Bio10 (Mean temperature of warmest quarter (°C)), Bio11 (Mean temperature of coldest quarter (°C)), Bio12 (Annual precipitation (mm)), Bio13 (Precipitation of wettest month (mm)), Bio14 (Precipitation of driest month (mm)), Bio15 (Precipitation seasonality (coefficient of variation)), Bio16 (Precipitation of wettest quarter (mm)), Bio17 (Precipitation of driest quarter (mm)), Bio18 (Precipitation of warmest quarter (mm)) and Bio19 (Precipitation of coldest quarter (mm)). Two environmental variables were also used for modeling i.e. altitude (m) and Normalized Difference Vegetation Index (NDVI). The first variable was derived from the Digital Elevation Model (DEM) of Iran with the same resolution, while NDVI was acquired from Aug 2014 image of MODIS satellite.

## Results

### Species Composition

Overall, 1143 mosquito larvae were collected including 6 species: *Anopheles maculipennis* s.l. (24), *An. superpictus* s.l. (4), *An. marteri* (10), *Culex hortensis* (454), *Cx. theileri* (429) and *Culiseta longiareolata* (222). The most and the least density was due to *Cx. hortensis* (39.72%) and *An. superpictus* s.l (0.35%), respectively (Table 1). *Anopheles maculipennis* s.l. was collected from both plain and foothill areas, but we could not find this malaria vectors in mountainous areas of Isfahan Province. *Culex theileri* and *Cx. hortensis* were the most aboundant species. Regardless of the topography of the area, they were caught in all the studied areas.

### MaxEnt Modeling

**Fig. 2.**
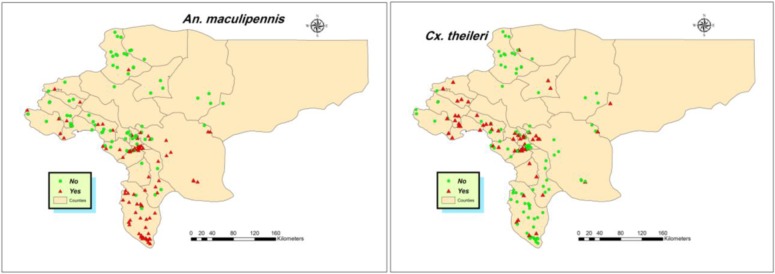
Spatial distribution of collection sites for *Anopheles maculipennis* and *Culex theileri* in Isfahan Province, Central Iran, 1984–2015

**Fig. 3.**
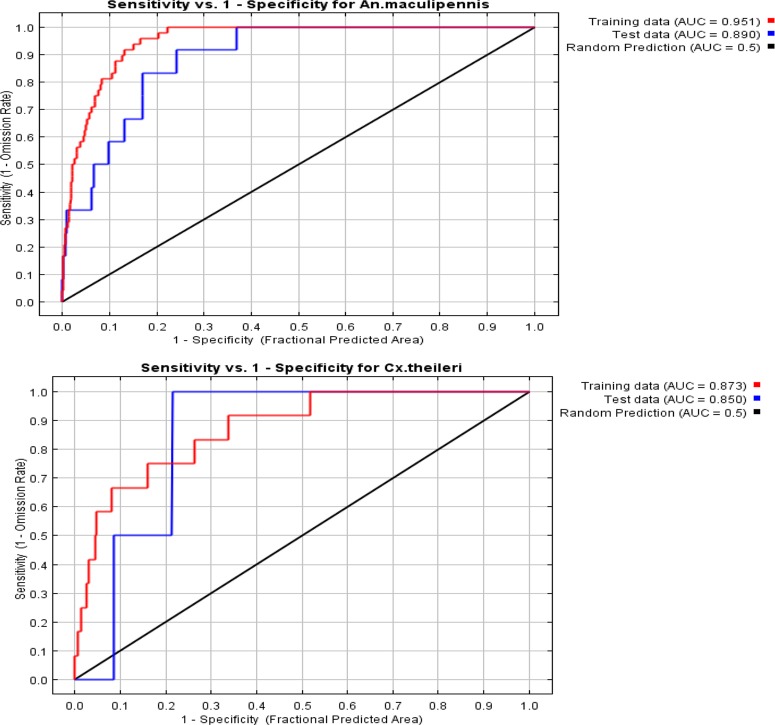
The area under ROC curve (AUC) for *Anopheles maculipennis* s.l. and *Culex theileri* in Isfahan Province, Central Iran, 2015

**Fig. 4.**
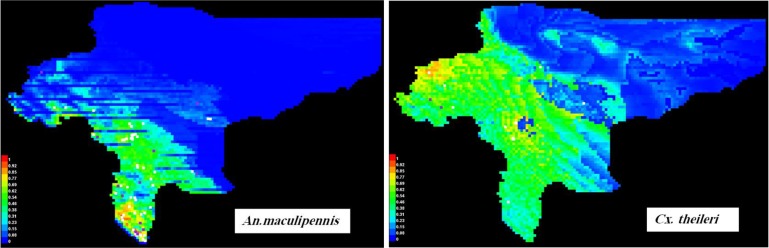
Probability of presence for *Anopheles maculipennis* s.l. and *Culex theileri* in Isfahan Province, Central Iran, 2015

This method was used to find the appropriate niches for *Cx. theileri* and *An. maculipennis* s.l. To do this, all collection points in this study, as well as earlier studies in Isfahan Province (1984–2015), were used to have enough occurrence data for modeling (Fig. 1). Figure 2 shows the collection sites for the studied species. The area under curve was calculated as 0.951 rather 1 for *An. maculipennis* (Fig. 3). More appropriate niches for *An. maculipennis* were restricted to the western and southwestern areas (Fig. 4). Based on jackknife test, the environmental variable with the highest gain when used in isolation is temperature seasonality (Bio4), which therefore appears to have the most useful information by itself. The environmental variable that decreases the gain the most when it is omitted is NDVI, which therefore appears to have the most information that is not present in the other variables. These numbers show an acceptable validity for the exported maps.

**Fig. 5.**
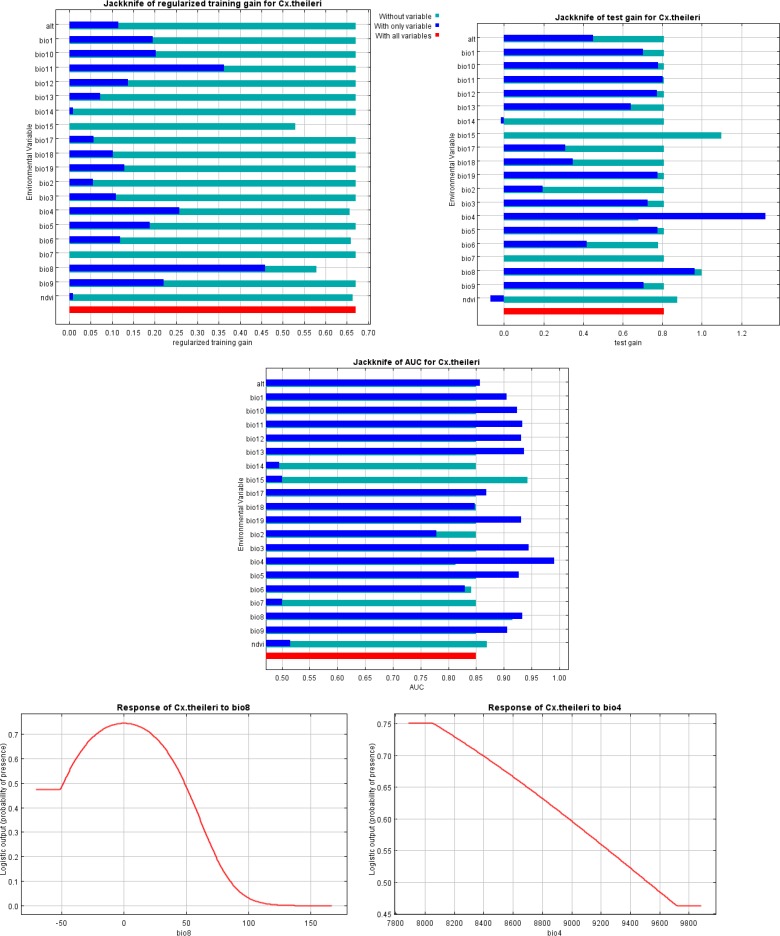
Jackknife test of AUC for *Culex theileri* in Isfahan Province, Central Iran, 2015

For *Cx. theileri*, the area under curve was calculated as 0.873 rather 1 (Fig. 3). Based on the maps, *Cx. theileri* had wider and more appropriate niches across the province comparing *An. Maculipennis* s.l., except for the eastern area (Fig. 4). Figure 5 shows the result of the jackknife test of variable importance for *Cx. theileri.* The environmental variable with highest gain, when used in isolation, was mean temperature of the wettest quarter (Bio8). This variable appears to have the most useful information for modeling this species. In Fig. 5 three jackknife plots for *Cx. theileri* were compared. The AUC plot shows that temperature seasonality (Bio4) is the most effective single variable for predicting the distribution of this mosquito species used for testing. The relative importance of temperature seasonality also increases in the test gain plot, when compared against the training gain plot. Mean temperature of wettest quarter is helping the model to obtain a good fit to the training data, but the temperature seasonality variable gives better results on the set-aside test data.

**Fig. 6.**
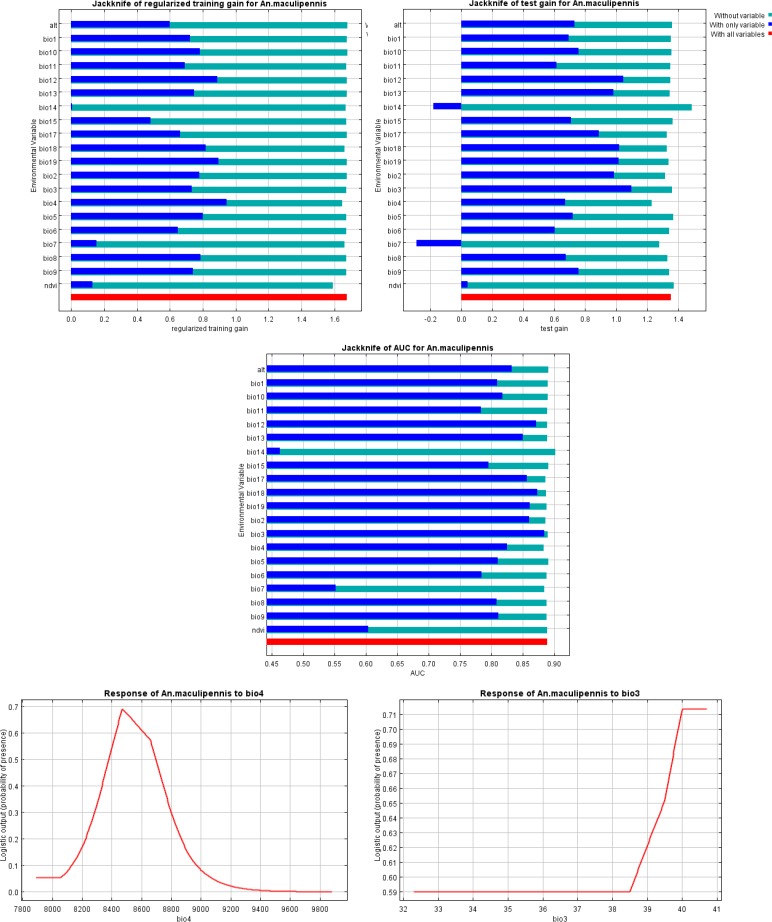
Jackknife test of AUC for *Anopheles maculipennis* s.l. in Isfahan Province, Central Iran, 2015

Figure 6 shows result of the jackknife test of variable importance for *An. maculipennis* s.l.. The environmental variable with highest gain, when used in isolation, was temperature seasonality (Bio4). This variable appears to have the most useful information for modeling this species. Comparing the three jackknife plots can be very informative. The AUC plot shows that isothermality (Bio3) is the most effective single variable for predicting the distribution of the occurrence data that was set aside for testing when predictive performance is measured using AUC, even though it was hardly used by the model built using all variables. The relative importance of isothermality also increases in the test gain plot, when compared against the training gain plot. Temperature seasonality is helping the model to obtain a good fit to the training data, but the isothermality variable gives better results on the set-aside test data.

**Table 1.** Spatial distribution of collected mosquito larvae according to topography and species, Isfahan Province, 2015

**County**	**Topography**	**Total Larvae (No.)**	No. and frequency of species											

			***An. maculipennis s.l***		***An. superpictus s.l.***		***An. marteri***		***Cx. hortensis***		***Cx. theieri***		***Cs. longiareolata***	

			**No.**	**%**	**No.**	**%**	**No.**	**%**	**No.**	**%**	**No.**	**%**	**No.**	**%**
**Khomeinishahr**	Plain	129	16	12.4	2	1.56	2	1.56	12	9.3	86	66.66	11	8.52
**Golpayegan**	Plain	50	4	8.3	2	4.2	0	0	6	12.5	36	75	0	0
**Mobarakeh**	Plain	65	0	0	0	0	0	0	33	50.77	32	49.23	0	0
**Najafabad**	Plain	90	0	0	0	0	0	0	57	61.95	35	38.05	0	0
**Faridan**	Foothill	115	4	3.47	0	0	8	6.96	0	0	34	29.57	69	60
**Khansar**	Foothill	247	0	0	0	0	0	0	152	61.54	42	17	53	21.46
**Fereidoonshahr**	Mountain	354	0	0	0	0	0	0	129	36.64	134	38.08	89	25.28
**Samirom**	Mountain	93	0	0	0	0	0	0	65	69.89	28	303.11	0	0
**Total**		**1143**	**24**	**2.1**	**4**	**0.35**	**10**	**0.88**	**454**	**39.72**	**429**	**37.53**	**222**	**19.42**

## Discussion

In this study six Culicidae mosquito species were collected and identified. Based on the previous studies in the province, 24 mosquito species are reported as follows: *Ae. vexans*, *An. algeriensis*, *An. claviger*, *An. dthali*, *An. maculipennis* s.l., *An. marteri*, *An. messeae*, *An. multicolor*, *An. sacharovi*, *An. superpictus*, *An. turkhudi*, *Cx. modestus*, *Cx. hortensis*, *Cx. mimeticus*, *Cx. perexiguus*, *Cx. pipiens*, *Cx. theileri*, *Cx. territans*, *Culiseta longiareolata*, *Cs. annulata*, *Cs. subochrea*, *Ochlerotatus caspius*, *Oc. pulcritarsus* and *Uranotaenia unguiculata* (16, 22, 23). Our findings report no new record for the province.

The dominant species in our study was *Cx. hortensis* (39.72%) followed by *Cx. theileri*. Earlier studies in Mobarakeh County found *Cx. theileri* as the dominant species in both larval and adult collections (23). Studies during 2003–2005 were also introduced *Cx. theileri* as the dominant species in Isfahan Province (24). Although in our study, the current species had the second density, it can be due to the number of collection times. On the other hand, if field sampling is done at a better time, a greater abundance may be due to this species. Generally *Cx. theileri* is a rice field mosquito species (28). It has usually exophilic and exophagic behavior and has a high anthropophilic index, and comprised more than 77% of the mosquitoes catched on human bait (21). Other studies were also reported *Cx. theileri* as the main species in some parts of Iran (29, 30).

*Culex theileri*, *An. maculipennis* and *An. superpictus* as three important vectors of parasitic agents to humans were collected in this study. In northwest of Iran, *Cx. theileri* infected was reported to *D. immitis* (7). Therefore, in addition to numerous bites on human in Isfahan area, it can be considered as probable vector of dirofilariasis. Among the collected mosquitoes, two main malaria vectors of Iran were identified: *An. maculipennis* and *An. superpictus*. In this study, *An. maculipennis* was only found in Khomeinishahr, Golpayegan and Faridan Counties. However, this species has reported from most counties of the province (23, 24). *Anopheles superpictus* was collected from Khomeinishahr and Golpayegan Counties in our survey, but there are reports of this species from 12 counties of the province (23, 24). There is potential for malaria transmission in the study area. Since the province has attractions and job opportunities, there are likely infected foreign immigrants from the endemic countries visit the area, at least in some months of the year. Three other species in this survey were *An. marteri*, *Cx. hortensis* and *Cs. longiareolata*. These species have no tendency to feed from human. Just *Cx. hortenis* known as birds malaria vector under laboratory condition (31). *Culiseta longiareolatsa* may rarely attack human and domestic animals, although there is little information on this species.

MaxEnt model was used to predict the distribution of some *Anopheles* mosquitoes around the world, such as *An. albimanus* in Americas (32), malaria vectors of Africa, Europe and Middle East (33), and *An. minimus* in southeast of Asia (34). A more recent study in Iran used this model to find the best ecological niches for three main malaria vectors in south of Iran, i.e. *An. stephensi*, *An. culicifacies* s.l. and *An. fluviatilis* s.l. (19). It is also used to predict the potential areas for *Cx. pipiens*, *Cx. tarsalis* and *Ae. vexans* in the United States and *Cx. tritaeniorhynchus* in Saudi Arabia (35, 36). AUC value in this research was 0.951 and 0.873 for *An. maculipennis* and *Cx. theileri*, respectively. Previous studies on MaxEnt model considered predicting AUC> 0.75 as good value for suitable niche for species (37). This suggests the MaxEnt prediction in our study is well. A new survey in Iran reported AUC values of 0.943, 0.974 and 0.956 for *An. stephensi*, *An. culicifacies* s.l. and *An. fluviatilis* s.l., respectively (19). In other countries AUC values were reported between 0.77 and 0.99 for different *Anopheles* species (36, 38–40). This difference is due to various ecological needs of these species.

## Conclusion

In addition to the problems caused by bites on humans, they can be considered for transmission of malaria and filariasis in the region. With due attention to tourist and business attractions of Isfahan Province, some people from different parts of the world will travel to different counties of the province annually. Considering the results of niche modeling for vector species, it is recommended to do additional studies on vectorial capacity of these species, their physiological age, and parasitic infection, and insecticide resistance susceptibility status to different insecticides to predict the risk of establishing the foci of filariasis, West Nile fever and malaria in Isfahan Province.
